# Age- and sex-specific deterioration on bone and osteocyte lacuno-canalicular network in a mouse model of premature aging

**DOI:** 10.1038/s41413-025-00428-x

**Published:** 2025-05-23

**Authors:** Dilara Yılmaz, Francisco C. Marques, Lorena Gregorio, Jérôme Schlatter, Christian Gehre, Thurgadevi Pararajasingam, Wanwan Qiu, Neashan Mathavan, Xiao-Hua Qin, Esther Wehrle, Gisela A. Kuhn, Ralph Müller

**Affiliations:** 1 Institute for Biomechanics, ETH Zurich, Zurich, Switzerland; 2 AO Research Institute Davos, Davos Platz, Switzerland

**Keywords:** Bone, Bone quality and biomechanics, Mitochondria

## Abstract

Age-related osteoporosis poses a significant challenge in musculoskeletal health; a condition characterized by reduced bone density and increased fracture susceptibility in older individuals necessitates a better understanding of underlying molecular and cellular mechanisms. Emerging evidence suggests that osteocytes are the pivotal orchestrators of bone remodeling and represent novel therapeutic targets for age-related bone loss. Our study uses the prematurely aged Polg^D257A/D257A^ (PolgA) mouse model to scrutinize age- and sex-related alterations in musculoskeletal health parameters (frailty, grip strength, gait data), bone and particularly the osteocyte lacuno-canalicular network (LCN). Moreover, a new quantitative in silico image analysis pipeline is used to evaluate the alterations in the osteocyte network with aging. Our findings underscore the pronounced degenerative changes in the musculoskeletal health parameters, bone, and osteocyte LCN in PolgA mice as early as 40 weeks, with more prominent alterations evident in aged males. Our findings suggest that the PolgA mouse model serves as a valuable model for studying the cellular mechanisms underlying age-related bone loss, given the comparable aging signs and age-related degeneration of the bone and the osteocyte network observed in naturally aging mice and elderly humans.

## Introduction

Age-related bone loss is a major health concern among older individuals characterized by decreased bone density, leading to increased susceptibility to fractures and compromised skeletal integrity, leading to diseases like osteoporosis and associated outcomes such as frailty^1–3^. While osteoporosis has long been associated with postmenopausal women, recent studies highlight its significant impact on men, an area that remains under-investigated^4,5^. Similarly, the literature indicates clear sex differences in frailty with females exhibiting greater frailty despite their lower risk of mortality compared to males^6–8^. Therefore, understanding the intricate cellular processes underlying these conditions is paramount since emerging evidence suggests that osteocytes represent a promising therapeutic target for mitigating age-related osteoporosis^9–12^.

Osteocytes, the most abundant cells and major mechanosensors in bone, play a crucial role in regulating bone remodeling^13,14^. These cells reside in small pocket-like spaces known as lacunae within the mineralized bone matrix and communicate through a complex network called the lacuno-canalicular network (LCN). In response to mechanical cues, osteocytes transmit signals that influence bone formation or resorption, thereby altering the bone microenvironment and regulating homeostasis. This mechanism is enabled through the gap junctions, secretory cell activities, and direct cell-to-cell contact via their dendrites^10,11,15–17^. Therefore, gaining insight into how osteocyte LCN changes within its native milieu during the aging process offers a deeper understanding of its pivotal role in age-related bone loss.

Advanced imaging techniques provided valuable insight into the age-related morphological changes in osteocytes and LCN in both humans and rodents. Micro-computed tomography (micro-CT) imaging allowed precise quantifications of the lacunar morphometry, illustrating a decline in lacunar density with aging on undecalcified bone samples^18–21^. Additionally, the assessment of osteocyte morphology and connections (connectomics) through confocal microscopy imaging combined with computational analysis showcased degenerations in the osteocyte and lacunar morphology with aging^22–26^. Hence, impairments in dendrites and LCN significantly compromise osteocyte function, thereby contributing to the age-related decline of bone.

An understanding of how aging is associated with changes in the osteocyte network may uncover new strategies to prevent age-related bone loss in older individuals. Our group has recently established the prematurely aged Polg^D257A/D257A^ (PolgA) mouse model to characterize age-related alterations in females^12,27^. The PolgA mice displayed a naturally accelerated aging phenotype and developed clinically relevant musculoskeletal aging characteristics, including kyphosis, alopecia, graying, and osteosarcopenia^28–30^. Yet the underlying mechanism driving the age- and especially the sex-associated changes in the osteocyte network remains unclear. Therefore, this study investigates these changes in bone and associated degeneration of the osteocyte network considering both age and sex factors in PolgA mice. We performed comprehensive musculoskeletal phenotyping that included frailty scoring, grip strength measurement, gait analysis, and bone morphometric analysis through ex vivo micro-CT. Subsequently, a detailed quantitative assessment of the osteocyte network was conducted in young and aged animals for both females and males. This analysis was facilitated by the application of FITC staining for undecalcified fresh frozen bone sections and multiphoton confocal imaging, complemented by a specialized new pipeline for in-depth in silico quantitative analysis. Given the key role of osteocytes in maintaining bone homeostasis^31^, a comprehensive understanding of the age-related deterioration of the osteocyte network is essential for unraveling the intricate mechanisms contributing to bone loss in older individuals.

## Results

### Age- and sex-specific musculoskeletal decline over time in PolgA mice

**Fig. 1:**
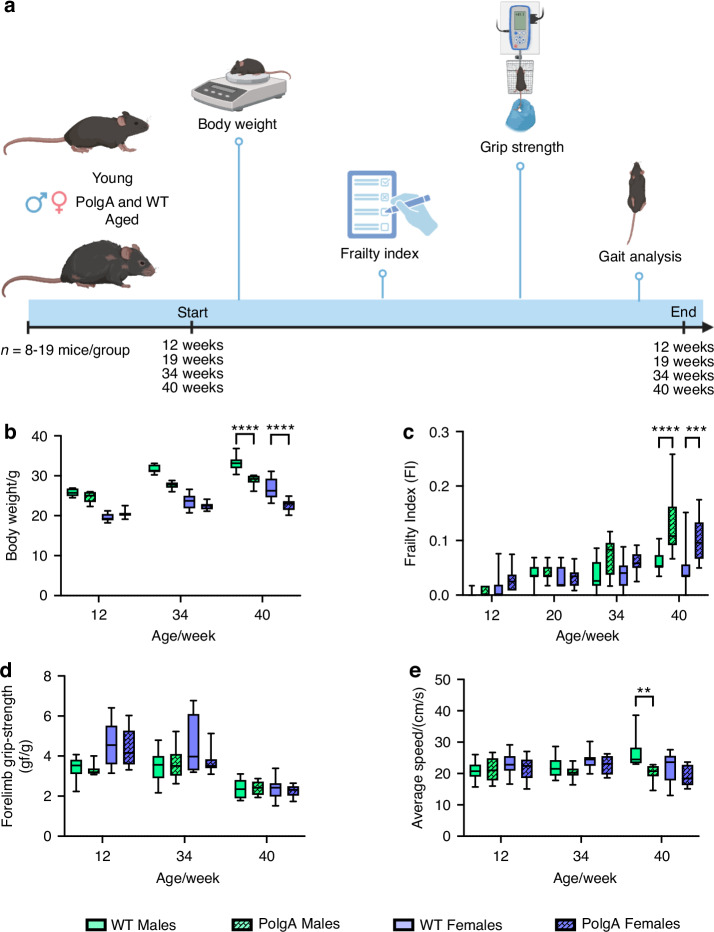
Musculoskeletal decline in PolgA mice with aging. **a** Schematic indicating the experimental plan (created with Biorender). Analysis of body weight changes (**b**), frailty index (FI) (**c**), forelimb grip strength (**d**) and average speed (**e**) over time in male and female PolgA mice and WT littermates. *n* = 7–13 mice/group. Significance was determined by two-way ANOVA with Tukey’s post hoc test (*P < 0.05, **P < 0.01, ***P < 0.001, ****P < 0.000 1)

To investigate the age-related changes in musculoskeletal phenotypes, we assessed the changes in body weight, clinical frailty index (FI), forelimb grip strength, and gait data analysis at 12, 19, 34 and 40 weeks for both male and female PolgA mice and their wild-type (WT) littermates, as indicated in Fig. 1a. Both mice gained weight progressively until 40 weeks, with males having a higher increase however at 40 weeks there was a significant decrease in the weight of PolgA mice compared to their WT counterparts for both sexes (males: –13% (*P* = 0.000 9), females: –15% (*P* = 0.173) (Fig. 1b). Similarly, both PolgA and WT cohorts demonstrated a notable progressive increase in FI with aging. At 40 weeks, however, PolgA males exhibited a significant increase of 52.30% (*P* < 0.000 1) in males and 61.53% in females (*P* = 0.000 2) compared to their WT littermates (Fig. 1c). Moreover, the grip strength of PolgA mice displayed a trend of reduced grip with age, although there was no significant difference between the genotypes. (Fig. 1d). Subsequently, a significant decrease in average walking speed was observed in PolgA mice compared to their WT counterparts, with males showing a 24% decrease and females experiencing a 15% decrease at 40 weeks (Fig. 1d). Concerning the remaining gait analysis parameters (Fig. S1A, B), we observed a 20.55% decline in stride length for the right hindlimb (RH) and a 15.80% decline for the left hindlimb (RH) and a 15.80% decline for the left hindlimb (LH) in male PolgA mice compared to their wild-type (WT) littermates at 40 weeks. (Fig. S1C). There was no significant difference in stride length for the right front^32^ limb between the groups; however, there was a 10.36% decline in stride length for the left front^22^ for PolgA males from 34 to 40 weeks (Fig. S1C). Likewise, a reduction in swing speed was observed in PolgA male mice from 12 to 40 weeks, showing an 8.75% decrease for the left front^22^ limb only. For PolgA females, there was a 19.45% reduction from 34 to 40 weeks, while the left hind (LH) limb experienced a 21.02% decrease (Fig. S1D). Reduced stride length^33,34^, indicates that the mice took shorter steps upon aging whereas decreased swing speed refers that the mice moved their limbs slower during the swing phase of locomotion. As stride length and swing speed decrease, aged PolgA mice might need to keep their feet on the ground longer to ensure balance and prevent falls, which may reflect a decline in motor coordination and agility. In contrast, neither male nor female PolgA mice did not exhibit an increased duty cycle in any of the limbs (Fig. S1E). Duty cycle is the percentage of the gait cycle that a foot is in contact with the ground^34^ (Fig. S1A, B), a decreased duty cycle refers to the feet of the mice spent less time in contact with the ground during each step. Hence, PolgA mice likely exhibited a greater decline in physical condition compared to their wild-type (WT) littermates at 40 weeks, with males showing a slightly more pronounced decline.

### Structural impairments in bone with age in prematurely aged PolgA mice

**Fig. 2:**
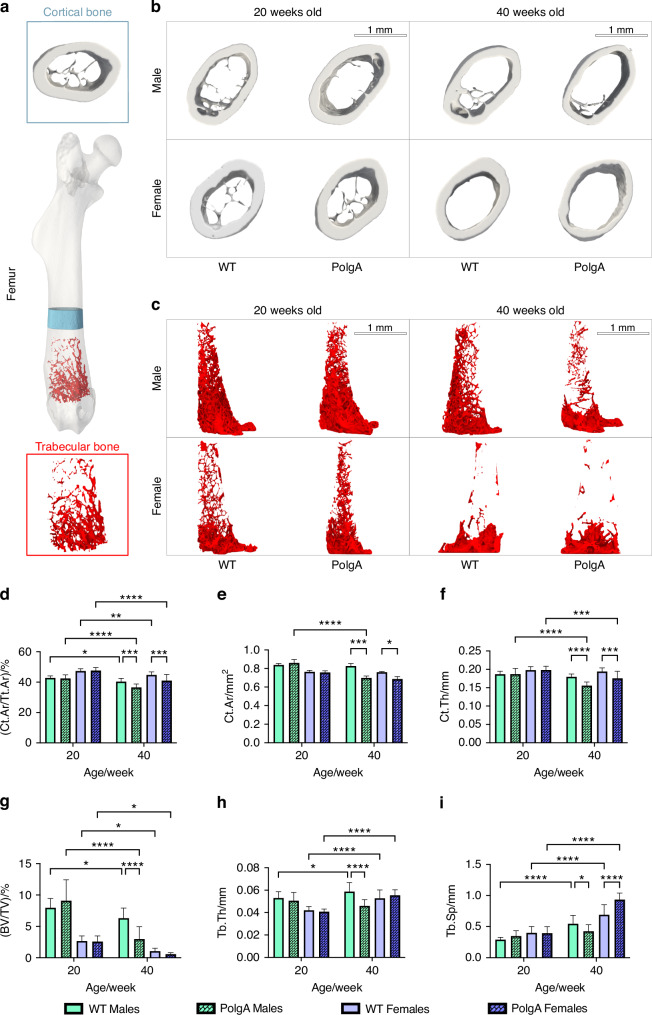
Age-specific structural impairments in bone in prematurely aged PolgA mice and WT littermates. **a** Representative 3D reconstructed longitudinal micro-CT image of right femurs showing cortical and trabecular bone regions used for analysis. **b** 3D cortical bone images of male and female mice at 20 and 40 weeks indicating the thinning of cortical bone and increased cortical bone area with aging. **c** 3D trabecular bone images of male and female mice at 20 and 40 weeks, showing the reduced trabecular bone volume with age in PolgA mice. **d**–**f** Decreased cortical bone parameters with aging in PolgA mice: cortical area fraction (Ct.Ar/Tt.Ar) (**d**), cortical area (Ct.Ar) (**e**), and cortical thickness (Ct.Th) (**f**). **g**–**i** Altered trabecular bone parameters with age in PolgA mice: bone volume fraction (BV/TV) (**g**), trabecular thickness (Tb.Th) (**h**) and trabecular spacing (Tb.Sp) (**i**). *n* = 8–16 mice/group. Significance was determined by two-way ANOVA with Tukey’s post hoc test (*P < 0.05, **P < 0.01, ***P < 0.001, ****P < 0.000 1)

To investigate the changes in the bone with aging, we performed micro-CT imaging on the right femurs of PolgA mice and their WT littermates at the ages of 20 and 40 weeks and evaluated standard morphometric parameters at each point. Micro-CT analysis revealed the changes in the structural composition of femurs with aging. Representative 3D reconstructed micro-CT images demonstrated a thinner cortical bone and reduced trabecular structure in PolgA mice at 40 weeks compared to their age-matched WT littermates and young counterparts (Fig. 2a–c, Sup Table 1). Bone morphometry analysis illustrated the notable changes in both trabecular and cortical bone. Cortical area fraction (Ct.Ar/Tt.Ar) was significantly reduced (9.50%) both in male (*P* = 0.001 3) and (8.31%) female (*P* = 0.026 5) PolgA mice in comparison to the WT littermates and young PolgA mice (males: 13.96% (*P* = 0.000 5); In PolgA mice compared to age- and sex-matched wild-type (WT) counterparts at 40 weeks, cortical area (Ct.Ar) showed a significant reduction, with males experiencing a 15.53% decrease (*P* = 0.003 1) and females a 9.51% decrease (*P* = 0.382). When compared to younger mice, the decrease was 18.88% (*P* = 0.028 9) in males and females at 9.39%. Similarly, cortical thickness (Ct.Th) was also reduced in aged PolgA mice compared to WT mice, with a 13.47% decrease in males (*P* < 0.000 1) and a 9.33% decrease in females (*P* = 0.024 9). This reduction was more pronounced when compared to younger mice, showing a 16.67% decrease in males (*P* = 0.000 8) and an 11.17% decrease in females (Fig. 2e, f). The decline in cortical parameters was observed to be more pronounced in PolgA males than females at 40 weeks.

In parallel to the observations in cortical bone, trabecular bone parameters were drastically altered in aged PolgA mice at 40 weeks (Fig. 2g–i). A particularly noticeable decreased trabecular bone volume fraction (BV/TV) in females was observed (Fig. 2g). With aging, BV/TV was decreased in PolgA mice for both sexes (males: 52.93% (*P* = 0.000 1), females: 48.03%); however, this difference was less apparent in females due to their lower trabecular density. Comparison of aged PolgA with their young counterparts indicated a 67.50 decrease in BV/TV in males (*P* = 0.030 0) and 79.28% in females (*P* = 0.015 3), Moreover, trabecular thickness (Tb.Th) was reduced (males: 20.69% (*P* = 0.000 1); females: 5.77%) while trabecular spacing (Tb.Sp) was decreased for males (22.28%) and increased for females (35.77%, *P* < 0.000 1). At 20 weeks, no differences were observed in bone phenotype and morphometric characteristics between PolgA and WT mice. However, at 40 weeks, PolgA mice showed compromised bone structure compared to age-matched WT littermates and young mice, without striking differences identified between males and females of the PolgA group (Fig. 2). Similarly, trabecular parameters showed a greater significant decline in PolgA males compared to females. At 40 weeks, the data indicated that female PolgA mice showed fewer significant differences from their WT littermates compared to male PolgA mice.

### Deterioration of osteocyte network in PolgA mice with aging

**Fig. 3:**
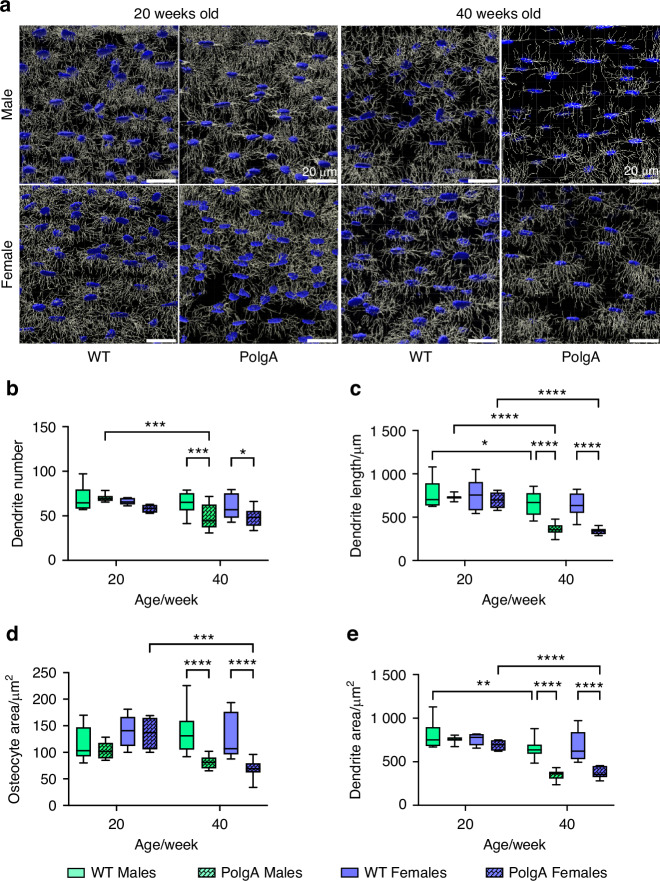
Age- and sex-related changes in osteocyte network of PolgA mice and WT littermates. **a** Representative 3D reconstructed Phalloidin-Hoechst stained images showing altered osteocytes and their dendrites with age in male and female PolgA mice using automated filament tracing in IMARIS. **b**–**e** Quantification of osteocytes and their dendrites from 3D Phalloidin-Hoechst image stacks of male and female mice indicating the degenerated osteocyte and dendrite features in PolgA mice: dendrite number (**b**), dendrite length (**c**), osteocyte area (**d**), and dendrite area (**e**). *n* = 3–4 mice/group, statistical significance was determined by non-parametric mixed effect analysis with Uncorrected Fischer’s LSD test, (P* < 0.05; P** < 0.01, P*** < 0.001)

To investigate the impact of aging on osteocyte morphology, we performed Phalloidin/Hoechst staining to label osteocytes and their dendrites and quantified the stained sections from confocal image stacks through Fiji (ImageJ) and IMARIS filament tracing software by adapting the implementation of neuronal filament analysis^35–37^. Through these approaches, we were able to analyze and evaluate the morphology and characteristics of osteocytes and their dendrites robustly and quantitatively. In PolgA mice, the quantification of confocal image stacks demonstrated a notable reduction in osteocyte network as they aged (Fig. 3a, S2A). At 40 weeks, both male and female PolgA mice exhibited reduced osteocyte connectivity (Fig. 3a). Furthermore, analysis of the confocal image stacks identified instances of disconnected osteocytes and empty cell bodies with only dendrites, predominantly in aged PolgA mice. Significant reductions were observed in the quantification of osteocytes and dendrites in an age- and sex-dependent manner in PolgA mice. Specifically, dendrite numbers decreased by 25.52% in males (*P* = 0.000 9) and 20.67% in females (*P* = 0.011 3) when compared to WT mice at 40 weeks. A similar pattern of decrease was noted in the dendrite area, with reductions of 46.27% in males and 44.14% in females (*P* < 0.000 1 for both). Dendrite length also exhibited similar trends, decreasing by 45.27% in males and 46.76% in females (*P* < 0.000 1 for both). Additionally, the osteocyte area decreased by 41.60% and 45.57% in males and females, respectively (*P* < 0.000 1 for both), compared to age-matched WT littermates and younger PolgA counterparts (Fig. 3b–e). While these deteriorations in osteocytes were more pronounced in PolgA males than in females, no significant changes were observed in their young counterparts.

**Fig. 4:**
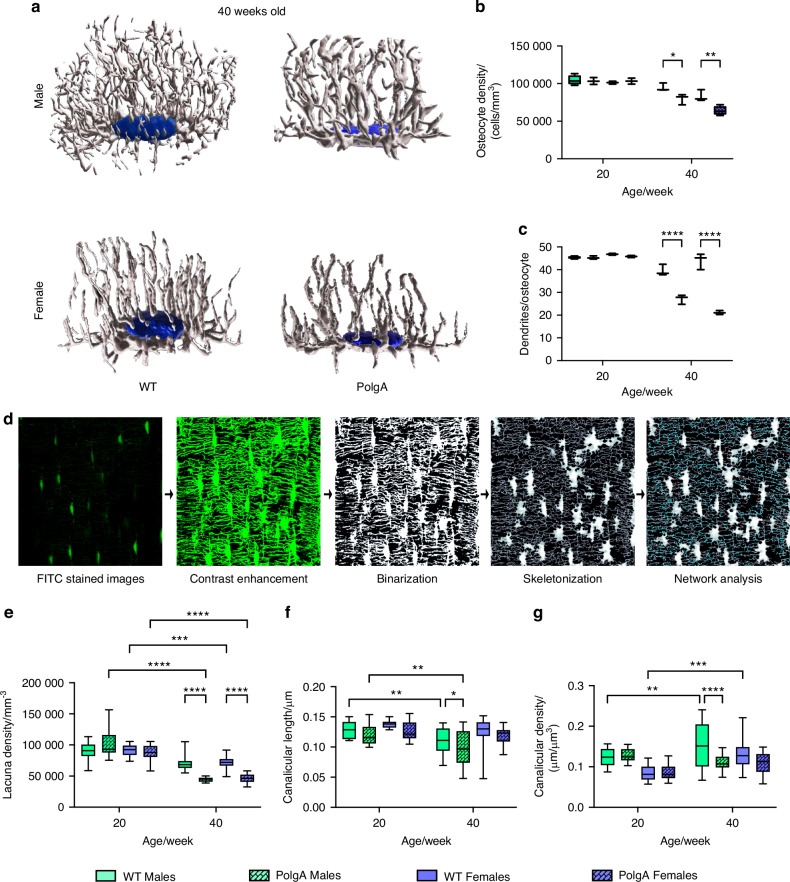
Degeneration of osteocyte and LCN in PolgA mice and WT littermates with age. **a** Representative 3D reconstructed Phalloidin-Hoechst stained images showing altered osteocytes and their dendrites with age in male and female PolgA mice 40 weeks. **b** Quantification of osteocyte showing the reduction in osteocyte density and (**c**) dendricity of male and female PolgA mice at 40 weeks. **d** Image processing workflow for the connectomics analysis of LCN showing, FITC stained confocal images, contrast enhancement, binarization, and skeletonization steps for osteocyte network analysis. **e**–**g** Quantification of LCN parameters showing degeneration with aging in PolgA mice at 40 weeks: lacuna density (**e**), canalicular length (**f**) and canalicular density (**g**). *n* = 3–4 mice/group. Statistical significance was determined by non-parametric mixed effect analysis with Uncorrected Fischer’s LSD test, (P* < 0.05, P** < 0.01, P***< 0.001)

Furthermore, a notable decline in the osteocyte density and dendricity (number of dendrites per osteocyte) obtained from the manual counting method was observed in PolgA mice (Fig. 4b, c). Osteocyte density decreased by 16% in males (*P* = 0.037 5) and 19% in females (*P* = 0.004 0), while the total number of dendrites reduced by 30.76% in males and 37.51% in females in PolgA mice compared to WT mice at 40 weeks. 3D images of osteocytes support the evident degenerative changes of osteocytes with aging in PolgA mice, illustrating the decrease in dendrites and an elongated and flattened osteocyte body (Fig. 4a).

### Disrupted LCN architecture and reduced connectivity within aging

For a more in-depth analysis of the osteocyte network, we performed FITC staining on undecalcified thick, optically cleared femur sections. Since Phalloidin specifically labels the actin filaments of the osteocytes, its visualization is restricted to the dendrites, whereas FITC can permeate the bone matrix and fill spaces like lacunae and canaliculi, thereby facilitating a detailed visualization of LCN. Therefore, by quantifying the FITC-stained images, we demonstrated the structural alterations of the LCN with aging (Fig. 4c–g, 4, S1B). LCN network in aging bone was sparser and less connected (Fig. S2) aligning with the findings in the aforementioned osteocyte analysis with Phalloidin-stained images.

**Fig. 5:**
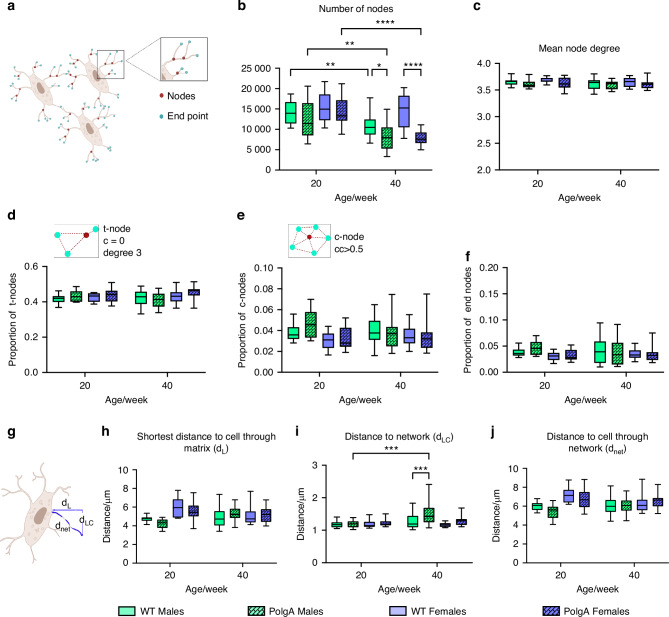
Disrupted LCN connectivity with aging in PolgA mice. Changes in the nodal analysis of the osteocyte network in PolgA mice and WT littermates showing classification of nodes from the connectomics analysis (**a**), and the number of nodes indicating the loss of network connection with aging (**b**). **c** Mean node degree (connections per node) showing the maintenance of network architecture. **d**–**j** Proportion of tree (t)-nodes (**d**), proportion of cluster (c)-nodes (**e**), proportion of end point nodes (**f**), illustration of the distance of the bone matrix from lacunae and the canalicular network (**g**), distance to the nearest cells through the matrix (**h**), the average distance to LCN from the matrix (**i**), and distance of the network from the next lacuna (**j**). *n* = 3–4 mice/group. Statistical significance was determined by non-parametric mixed effect analysis with Uncorrected Fischer’s LSD test, (P* < 0.05, P** < 0.01, P***< 0.001)

Lacuna density was significantly reduced in PolgA mice compared to their controls at 40 weeks (Fig. 4e). Moreover, the shortening of canaliculi and decline in the canaliculi density were apparent in aged PolgA mice in comparison to their littermates and young counterparts (Fig. 4f, g). Moreover, using connectomics analysis^24,25^, we quantified nodes in addition to a 3D osteocyte network of young and aged PolgA compared to WT littermates (Fig. 5). The mean number of nodes decreased with aging for both PolgA and WT mice, however; this reduction was greater in the PolgA group compared to controls and young mice, indicating the loss of network interaction in aging (Fig. 5b). In contrast, the mean node degree, showing the number of connected nearest neighbors remained similar across all groups, suggesting that the network is preserved at a similar level despite the changes in the osteocyte connectivity (Fig. 5c). The proportion of all node types: tree nodes (t, c = 0), cluster nodes (c, cc >0.5), and end nodes remained constant among all groups indicating the overall robustness and resilience of network architecture with different age groups (Fig. 5d–f).

Furthermore, distances within the LCN network were quantified. Aged bone displayed lower canalicular length, resulting in increased molecule travel distance from the extracellular matrix (ECM) to LCN, thereby d_LC_ was greater significantly in aged PolgA mice compared to their respective controls and young mice, while other variables describing the distance to the nearest cell directly through the matrix (d_L_) and the distance within the LCN to the nearest lacuna through the network (d_net_) remained unaffected by the age-related alteration in LCN (Fig. 4g–j). Hence, analysis of LCN features revealed degenerative alterations in aged PolgA mice when compared to aged-matched WT littermates at 40 weeks, as well as in young PolgA mice, indicating degenerative changes in the osteocyte network of PolgA mice with age.

## Discussion

In this study, a detailed analysis was conducted to investigate the age- and sex-related changes in the musculoskeletal health parameters, bone and osteocyte network in prematurely aged PolgA mice. Our findings revealed that the PolgA mice demonstrated significant age-related impairments at 40 weeks compared to their WT littermates and young counterparts. These mice exhibited musculoskeletal physical decline characterized by weaker grip strength, reduced speed, increased frailty, and body weight loss as they aged, more prominently observed in males. Despite the slightly higher frailty observed in females, other parameters such as reduced mobility were more pronounced in aged PolgA males^38^. This discrepancy may stem from the greater increase in the frailty index observed in females, while males exhibited more physical decline by 40 weeks. Considering 40 weeks is not typically recognized as old for a mouse, PolgA mice displayed signs of aging as early as this age. This is consistent with the findings from aged mice roughly at 23 months and approximately 75-year-old humans^12,39,40^. The changes in their musculoskeletal health parameters were followed by the changes in their bone and osteocytes. PolgA mice at 40 weeks demonstrated increased cortical diameter and area fraction, diminished cortical thickness, and a reduction in trabecular bone volume and thickness. Likewise, male PolgA mice exhibited more deteriorated bone structure than females. In particular, our data demonstrated the degeneration of the osteocytes and their dendrites in PolgA mice, with more prominently observed in males using the automatic in silico quantification methods. Furthermore, we illustrated the disrupted LCN connectivity with aging in PolgA mice by employing the combination of FITC staining for fresh frozen sections and a newly developed connectomics analysis. Hence, we showcased the degeneration of osteocytes LCN and bone in prematurely aged PolgA mice.

Furthermore, at 40 weeks, age-related alterations in the bone of PolgA mice closely resembled those observed in both aged C57Bl/6 mice and humans^41–44^. However, in C57BL/6 mice, age-related changes were reported to be more pronounced in females exhibiting a greater loss in trabecular bone and increased cortical porosities compared to males^41^.

Consistent with previous findings, aging alters the osteocyte network in PolgA mice where the osteocyte density and dendricity were reduced at 40 weeks similarly observed in C57Bl/6 mice at 22–23 months and in aged humans at 60–75 years^26,45,46^. However, deteriorations of the osteocyte network were observed to be slightly more pronounced in aged male PolgA mice rather than females evident in the 3D images as well (Fig. S2), contrasting to the findings in C57Bl/6 mice and humans. This could be attributed to the presence of estrogens in female PolgA mice at 40 weeks, potentially providing a protective benefit to mitochondria and alleviating the age-related effects of POLG mutations to a greater degree compared to males.

Additionally, through semi-automated quantification with IMARIS, we successfully captured detailed information on the degeneration of dendrites and osteocytes. IMARIS filament tracing method, originally designed for 3D tracking and high throughput analysis of neurons, was adeptly repurposed for osteocyte cell counting and dendrite quantification due to its precise object detection and accurate morphological analysis^35,47–49^. This approach allowed for robust and in-depth evaluation while obtaining additional information about osteocytes and their dendrites, including dendrite volume, area and length, and osteocyte area^36^.

Moreover, we tailored the FITC staining for fresh frozen undecalcified sections in order to perform connectomics analysis. This enabled the high-contrast labeling of the bone, which was further enhanced with 2,2′-thiodiethanol (TDE) optical clearing and multiphoton confocal imaging. This combination minimizes imaging artifacts and enhances resolution, allowing precise identification of canaliculi within the deep mineralized bone matrix without requiring tedious sample processing with acid-etching of resin-embedded bone or in vivo dye injections^23,46,50–52^. Also, the challenges associated with visualizing the 3D network in 2D paraffin sections stained for LCN were overcome through connectomics analysis applied to FITC-stained 3D image stacks. In addition, we have developed a new image-processing pipeline for connectomics analysis. This pipeline enables a reproducible segmentation of the LCN, whose morphometry can be analyzed with existing evaluation methods (e.g., cschurm/ocy_connectomics_updated)^22^. It provides a reliable identification of the LCN and is sensitive to differences between PolgA and WT groups in LCN morphometric parameters.

Additionally, we compared cell numbers with traditional hand-counted data using IMARIS and/ or Fiji (Cell counter plugin) against our in-house developed connectomics pipeline based on Python. For the aged male group, the mean cell number was 25.41 ± 5.87 for manual counting and 32.95 ± 7.91 for the automated pipeline. The respective standard error of the mean is 0.94 and 1.26. The mean percentage of error is 33.20%, with a standard deviation of 34.31. Significant discrepancies between these methods were confirmed via a Wilcoxon matched-pair rank test (*P* < 0.000 1) (Fig. S3A). Moreover, a Bland-Altman test was performed to evaluate the methodological agreement, revealing a mean difference of -7.52 (Fig. S3B). These differences were anticipated, as our manual counting method intentionally excludes cells at the edges of images due to their potential incompleteness and lower reliability. Conversely, the automated Python pipeline counts all visible cells, including those at the edges, typically inflating the count. Despite the inherent differences in methodological approaches, our automated pipeline is still sensitive enough to detect differences within aging. Moreover, the robustness of our findings is supported by consistent results obtained by different observers. These observers have independently confirmed the conclusions on various parameters. These findings highlight the reliability of both manual and automated counting methods, and these analyses not only clarify the operational discrepancies between manual and automated cell counting but also validate the efficacy of automated methods in capturing significant biological variations, which is crucial for aging research. This underscores the utility of the automated pipeline in extensive studies where manual counting would be impractical or unfeasible, although care must be taken in interpreting results due to methodological biases.

Furthermore, connectomics analysis revealed the canalicular loss and degeneration of LCN with aging in PolgA mice, akin to the findings in aged C57Bl/6 mice and mice with disrupted TGF-b pathway^25,53^. Their findings emphasized the loss of canaliculi with aging without taking into account the changes in the lacunae that were consistent with earlier findings, where no reduction in the number of lacunae was observed, thus underlining the significant role the canalicular network in osteocyte function^25,54–56^. However, in our study, we briefly assessed the changes in the lacunae as well to underscore that alterations in both the canaliculi and lacunae contribute to osteocyte function and overall bone health. Moreover, their study highlights age and sex differences in aged WT mice, showing that TGFβ signaling declines with age, impairing osteocyte function and leading to bone aging, particularly in males^53^. Bone mass and density changes occur independently, while young females maintain bone quality without TGFβ signaling, though this effect weakens with age. Overall, their data indicate that osteocytes are key to age-related declines in bone quality^53^.

Furthermore, our connectomics analysis exhibited impaired LCN architecture in PolgA mice at 40 weeks, with reduced lacunar density, canalicular density, and length, similar to the findings from aged mice and humans^32,57–59^. On the other hand, the nodal analysis of PolgA mice showed a similar extent of changes as observed in the aged WT mouse bone^25^. This could be attributed to the comprehensive nature of our connectomics analysis, including a larger region to quantify osteocyte networks than the previous publications^22,24,25^. While our study did not particularly focus on predicting fluid mechanics or investigating mechanotransduction of osteocytes concerning the age-related changes in LCN, it is possible to achieve these by combining confocal imaging of osteocyte LCN with connectomics analysis and further applying 3D finite element (FE) modeling^60–63^. Additionally, we have experimented with imaging FITC stained sections using confocal microscopy before multiphoton microscopy, both with and without TDE and our current approach has yielded the best results thus far. Hence in silico approach for the analysis of osteocyte networks offers a unique opportunity to overcome experimental challenges in investigating the connection between osteocyte and LCN structure.

Although PolgA mice do not fully capture the complex relationship between LCN degeneration leading to osteocyte death and the subsequent bone deterioration caused by osteocyte loss, our study with this model reveals the age- and sex-specific degeneration of bone, osteocyte, and LCN as early as 40 weeks, similar to those observed in aged mice and humans. Future studies should incorporate natural aging models to compare and validate the findings obtained from the PolgA model. Consequently, our findings suggest that the PolgA mouse model could serve as a robust model for investigating the molecular mechanisms responsible for age-related bone loss. Furthermore, it underscores the critical contribution of osteocytes to bone remodeling and health.

## Materials and methods

### Animal model

All animal procedures adhered to the regulations established by local authorities and received approval from the Veterinäramt des Kantons Zürich, Zurich, Switzerland (license: ZH35/2019). A mouse colony expressing an exonuclease-deficient version of the mitochondrial DNA polymerase γ (PolgAD257A, B6.129S7(Cg)-Polgtm1Prol/J, JAX stock 017341, The Jackson Laboratory) was bred and maintained at the ETH Phenomics Center. The mice were housed under standard conditions, featuring a 12:12 h light-dark cycle, access to ad libitum maintenance feed and water, and three to five animals per cage following previously outlined protocols^27^. Genotyping was performed before experiments by Transnetyx (Cordova, USA). Homozygous PolgA mice, along with age- and sex-matched wild-type (WT) littermates (controls), were used for all experiments^64^.

### Quantification of frailty index

The Frailty Index (FI) was quantified at 12, 19, 34 and 42 weeks of old PolgA mice and their WT littermates for both sexes using the mouse clinical Frailty Index (FI). This consists of a comprehensive evaluation of 31 non-invasive clinical parameters^27,65,66^. Each parameter was assigned a score of 0 for absence, 0.5 for a mild deficit, and 1 for a severe deficit except for body weight which was scored relative to the standard deviations from a reference mean in young adult mice (12 weeks old)^67^. Body surface temperature was excluded from the calculation of the FI due to the unavailability of suitable measurement tools.

### Grip strength

Grip strength was evaluated using a force tension apparatus (Grip Strength Meter, model 47200, Ugo Basile) at 12, 19, 34 and 40 weeks in female and male PolgA mice and WT littermates as previously described^27^. The mice grasped a stationary bar with their forepaws and were subsequently pulled horizontally by their tail until they released their grip. From the five measurements taken for each mouse, the maximum force (in gram-force) value was recorded and used for the analysis. The same trained operator performed all grip strength measurements.

### Gait analysis

Gait data was conducted on both female and male PolgA and WT mice 12, 19, 34 and 40 weeks of age. Noldus CatWalk XT system was utilized to analyze several critical gait parameters—run speed, standing duration, duty cycle, swing phase speed, maximum variation, and stride length—to thoroughly assess the gait characteristics of the mice^3^.

### Micro-CT image analysis

Right femurs were fixed for 4 h in 4% PFA at 4 °C and were subjected to micro-CT scanning in PFA (micro-CT 45, Scanco Medical AG) with an isotropic voxel-size of 10 μm; 55 kVp, 145 μA, 200 ms integration time. A Gaussian filter (support: 1) with a sigma of 0.8 was applied to 3D image data and segmented with a threshold of 392 mgHA/cm^68^. Cortical and trabecular compartments were identified following the previously described method^69^. Standard bone morphometric parameters were evaluated (Table S1).

### Bone immunohistochemistry

As previously described, femurs harvested from 20 to 40-week-old PolgA mice and their age-matched WT littermates were fixed immediately in ice-cold 4% paraformaldehyde (PFA) for 24 h at 4 °C. Right femurs were decalcified in 12.5% EDTA for 10-11 days at 4 °C, and left femurs were undecalcified. Bones went through overnight incubation in sucrose solution (20% sucrose, 2% PVP) and embedded in OCT. Samples were stored at −80 °C until sectioning. 50 μm-thick longitudinal cryosections of full bone (Thermo-Fischer Scientific, CryoStar NX70) were obtained at a standardized location using MX35 premier disposable low-profile blades (Epredia)^64^. No section polishing after cutting was performed.

Bone sections were hydrated with PBS three times at 5 min intervals, followed by permeabilization with 0.3% Triton X-100 in PBS for 20 min at room temperature (RT)^21^. Sections were immersed in blocking solution (5% bovine serum albumin (BSA) in 0.025% Triton X-100) for 45 min at RT. Following the sections were co-incubated with Phalloidin conjugated Alexa Flour 555 (ab176756, Abcam, 1:1 000) and Hoechst (94403, Sigma Aldrich, 1:500) in 0.3% BSA in PBS for 1 h to stain the actin cytoskeleton. Sections underwent a triple wash in PBS at 10 min intervals, followed by air-drying and, mounting with Prolong Diamond antifade mountant (P36961, Thermo Fischer), and the edges were sealed with nail polish.

For FITC staining, undecalcified bone sections were hydrated with gradually increasing concentrations of EtOH (75%, 95% and 100%) for 5 min each on a shaker and then incubated with a freshly prepared 0.01% fluorescein isothiocyanate isomer I (FITC) solution (F7250, Sigma Aldrich) prepared in 100% ethanol (#100983101, Supelco) 2 days in the dark. The FITC solution was renewed, and the sections were further incubated for an additional 3 days on a shaker in the dark. Subsequently, the sections were washed with 100% ethanol three times for 15 min each, followed by air-drying. Next, sections were hydrated with PBS for 5 min twice. Following rehydration, the sections were optically cleared with a gradually increasing concentration of 2,2’-thiodiethanol (TDE) (166782, Sigma Aldrich) for 2 h each, followed by an overnight incubation in a 97% TDE solution. Finally, the sections were mounted in 97% TDE, and the edges were sealed with nail polish.

The right femurs, which were scanned using micro-CT, underwent a decalcification process and were then used for staining with Phalloidin and Hoechst. On the other hand, the left femurs were not decalcified and were used for staining with FITC.

### Image acquisition and quantitative analysis

All bone sections were consistently sectioned in the same manner and maintained in the same orientation during imaging to ensure uniformity and reproducibility of the results. We imaged both distal and midshaft regions for all staining. However, for the purposes of this manuscript, we focused primarily on 6–10 regions per mouse around the midshaft. This decision was made because, following FITC staining, some bone sections disintegrated, particularly in the distal regions. Additionally, images from the distal regions were often less uniform compared to those around the midshaft, limiting us to max about 4 usable regions from the distal side for FITC staining. Phalloidin-Hoechst stained sections were imaged on midshaft and distal regions with Leica SP8 confocal microscopy while FITC stained sections were imaged on midshaft region with Leica SP8 MP multiphoton microscopy with the dimensions of 0.2 μm × 0.2 μm. Z-stacks of images were processed and 3D-reconstructed using IMARIS (Oxford Instruments). The filament tracer tool in IMARIS was utilized for the reconstruction and quantification of dendrites. Consistent threshold parameters were applied to both PolgA and WT mice across all age groups and sexes. Dendrite area, volume, length, and osteocyte area were used for statistical analysis. Additionally, osteocyte number, dendrite number, osteocyte area, and density were quantified using Fiji for comparison with IMARIS. Output from IMARIS was used for this paper. Image processing steps comply with Nature*’s* Guide for digital images.

Prior to connectomics network analysis, images were pre-processed in Python (Python 3.11.6, scipy 1.10.1, scikit-image 0.20.0) to enhance the details of the LCN network. First, the brightness of each section in a z-stack was adjusted using a Python implementation adapted from Fiji’s “Auto brightness correction” function in a sliding-window approach (kernel: 50 µm and stride: 8 µm), followed by Gauss-filtering to reduce noise (sigma determined as: 3.25 x pixel size in µm). After intensity normalization, contrast-limited adaptive histogram equalization was applied (kernel: 6 µm) to enhance lacunae and canaliculi details. Subsequently, contrast adjustment based on sigmoid correction was performed with an image-specific cut-off value that scaled the range of the intensity histogram to the interval (0, 1). Finally, images were rescaled to an isotropic resolution of 0.4 µm and binarized (threshold: 0.6). Images were analyzed in MATLAB (MATLAB 2022a, Mathworks), as described previously^22^. An initial random subset of 5 images was used to calibrate the health parameters of the MATLAB connectomics analysis, based on visual inspection of the lacunae and canaliculi identified and comparison to the number of lacunae counted manually. For subsequent analysis of the entire dataset, the lacunae identification values were set as distance threshold to identify potential lacunae as 5, minimum object size in voxels as 25, distance threshold for dilation as 2, and limit fraction for dilation as 0.1.

### Statistical analysis

Data analysis was performed with GraphPad Prism software. Significant differences were determined with two-way ANOVA with Tukey post hoc for musculoskeletal health parameters and bone morphometry evaluation. 7-16 mice per group were used for analysis and data represented as mean ± s.d. *P* < 0.05 was considered significant. For osteocyte and LCN analysis, 3–4 mice per group used, with 6–10 regions per mouse for each section. Significant differences were determined using the nonparametric mixed effect analysis with a significant threshold set at *P* < 0.05. For the comparison of manual and automated cell counting methods (aged male groups), the mean standard deviation and mean percentage error were calculated, along with a Bland-Altman analysis, all performed using R (https://www.r-project.org/). Wilcoxon matched-pairs signed-rank test was performed in GraphPad to test for statistical differences, for 39 paired samples per group.

## Supplementary information

Supplemental Material Figures and Table

Supplemental Material Videos

Video S1. Visualization of lacuno-canalicular network (LCN) in male 40-week-old wild-type mouse

Video S2. Visualization of lacuno-canalicular network (LCN) in male 40-week-old PolgA mouse

Video S3. Visualization of lacuno-canalicular network (LCN) in female 40-week-old wild-type mouse

Video S4. Visualization of lacuno-canalicular network (LCN) in female 40-week-old PolgA mouse

Supplemental Material Python Code

## Data Availability

The authors declare that all data supporting the findings of the study are available in this paper and could be shared upon reasonable request from the corresponding author.

## Code availability

The MATLAB code is available in GitHub: https://github.com/phi-max/OCY_connectomics^22^. The python code for preprocessing the confocal images for connectomics analysis is available as Supplemental Material in this paper.
