# Supplementary material for: Age- and sex-specific deterioration on bone and osteocyte lacuno-canalicular network in a mouse model of premature aging
Source: Bone Res. 2025 May 23;13:55. doi: 10.1038/s41413-025-00428-x (PMC12102221; doi:10.1038/s41413-025-00428-x)
Supplement: Supplementary file 1 — Supplemental Material Figures and Table [file 41413_2025_428_MOESM1_ESM.docx]

**Supplemental Material**

**Age- and Sex-Specific Deterioration on Bone and Osteocyte Lacuno-Canalicular Network in a Mouse Model of Premature Aging**

Dilara Yılmaz^1^, Francisco C. Marques^1^, Lorena Gregorio^1^, Jérôme Schlatter^1^, Christian Gehre^1^, Thurgadevi Pararajasingam^1^, Wanwan Qiu^1^, Neashan Mathavan^1^, Xiao-Hua Qin^1^, Esther Wehrle^1,2^, Gisela A. Kuhn^1^, Ralph Müller^1*^

^1^Institute for Biomechanics, ETH Zurich, Zurich, Switzerland

^2^AO Research Institute Davos, Davos Platz, Switzerland


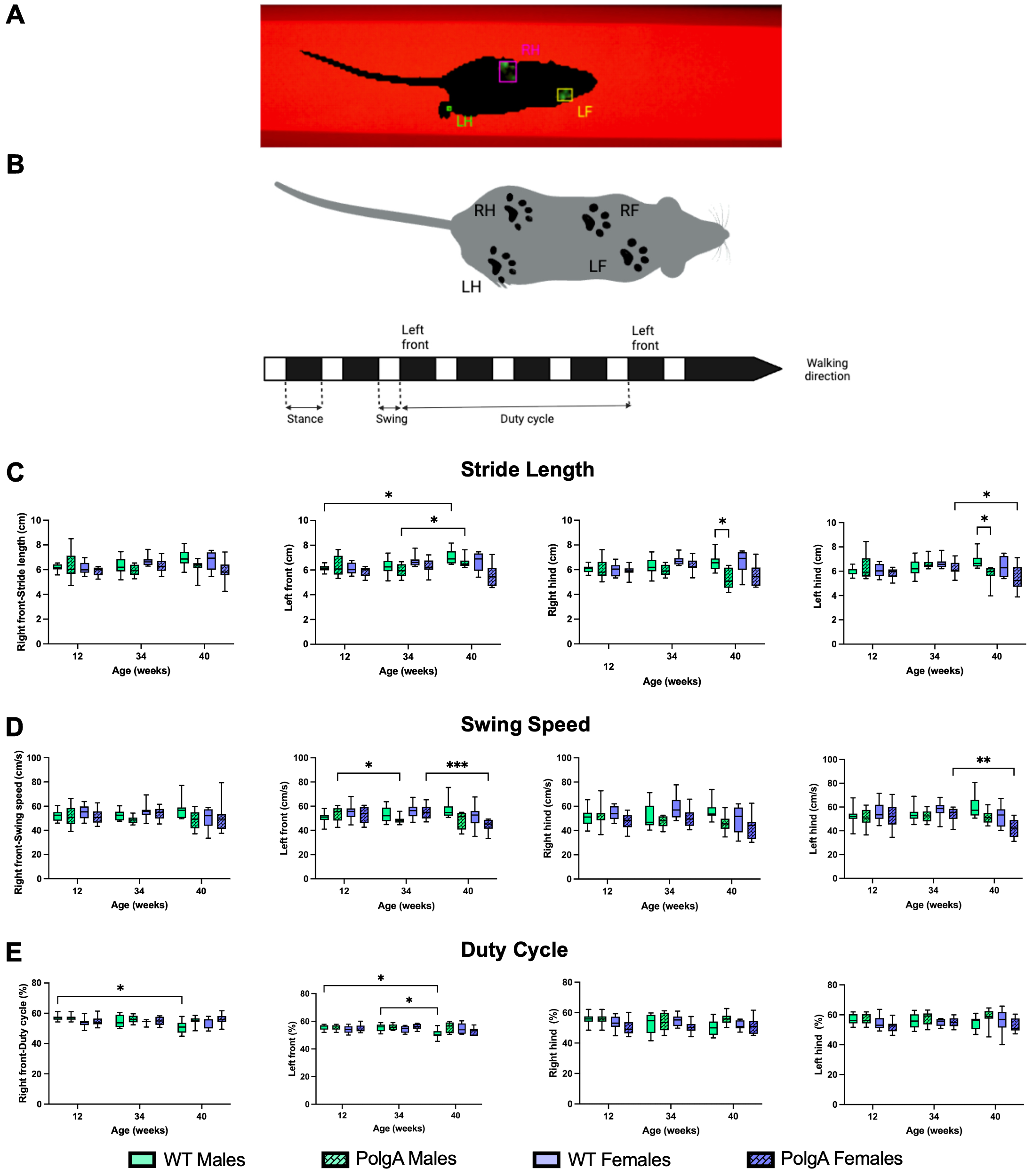


**Fig. S1:** Analysis of the age- and sex-related changes in the gait parameters in PolgA mice and WT littermates. A) Schematic illustration of the CatWalk gait analysis, capturing the camera footage of illuminated footprints on a glass plate. B) Illustration of CatWalk parameters, showing the stance, swing, and duty cycle (created with Biorender and adopted from Knox-Concepcion et al., Int. J. Mol. Sci. 2019, 20(14), 3493; doi.org/10.3390/ijms20143493) C) Stride length D) Swing speed (cm/s) E) Duty cycle (%) for each limb of male and female PolgA mice and their WT littermates across the different ages. (n=7-13 mice/group). Statistical significance was determined by two-way ANOVA with Tukey post hoc test (p*<0.05, **p<0.01, ***p<0.001).

**
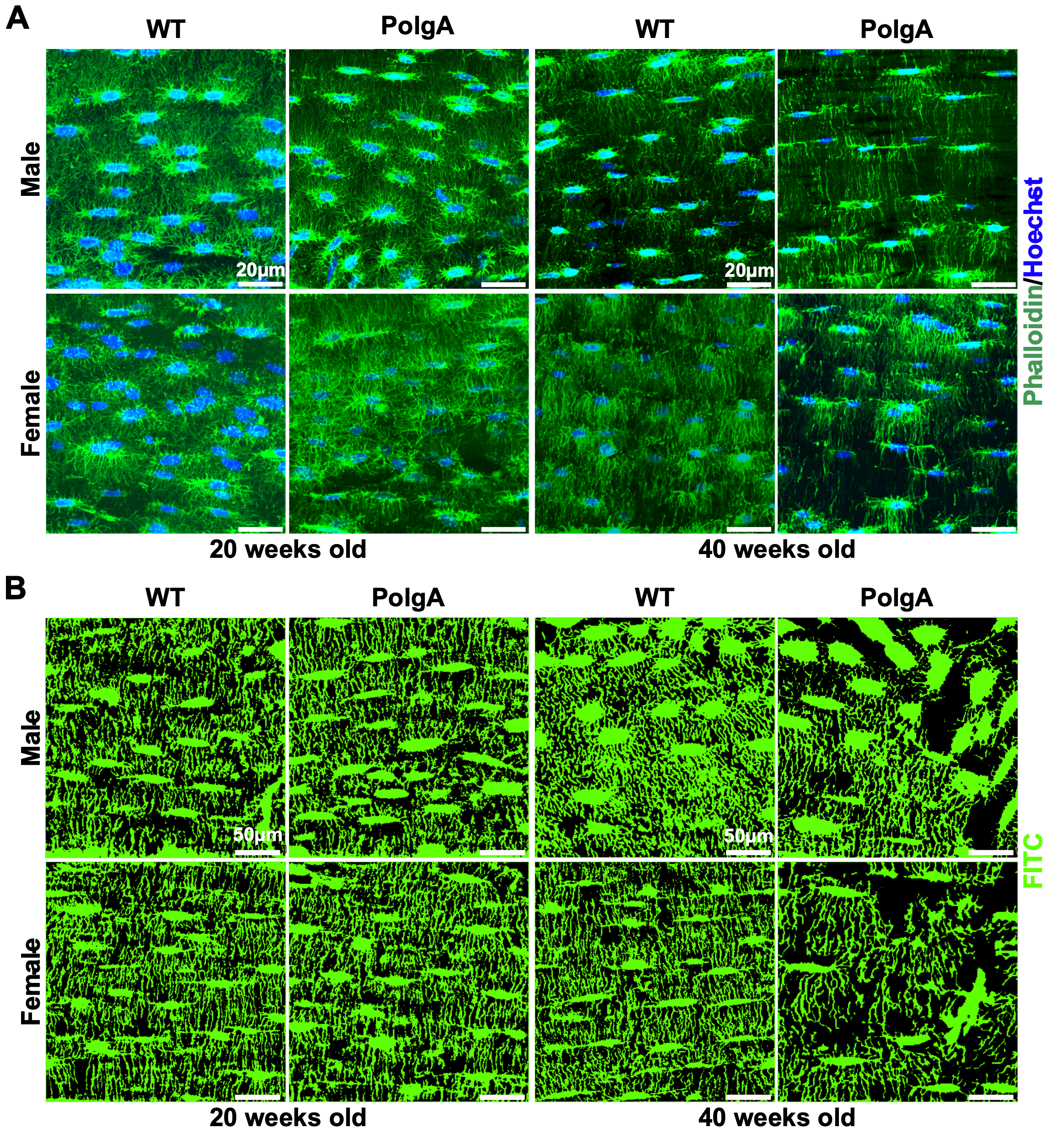
**

**Fig. S2:** Degeneration of osteocyte network and LCN connectivity with aging in PolgA mice and WT littermates. A) Representative high magnification images of Phalloidin (F-actin, green) and Hoechst (nucleus, blue) stained sections illustrating degeneration of osteocyte networks and B) representative maximum intensity projection images of FITC stained sections showing LCN disruption in male and female PolgA mice and their WT littermates at 20 and 40 weeks.


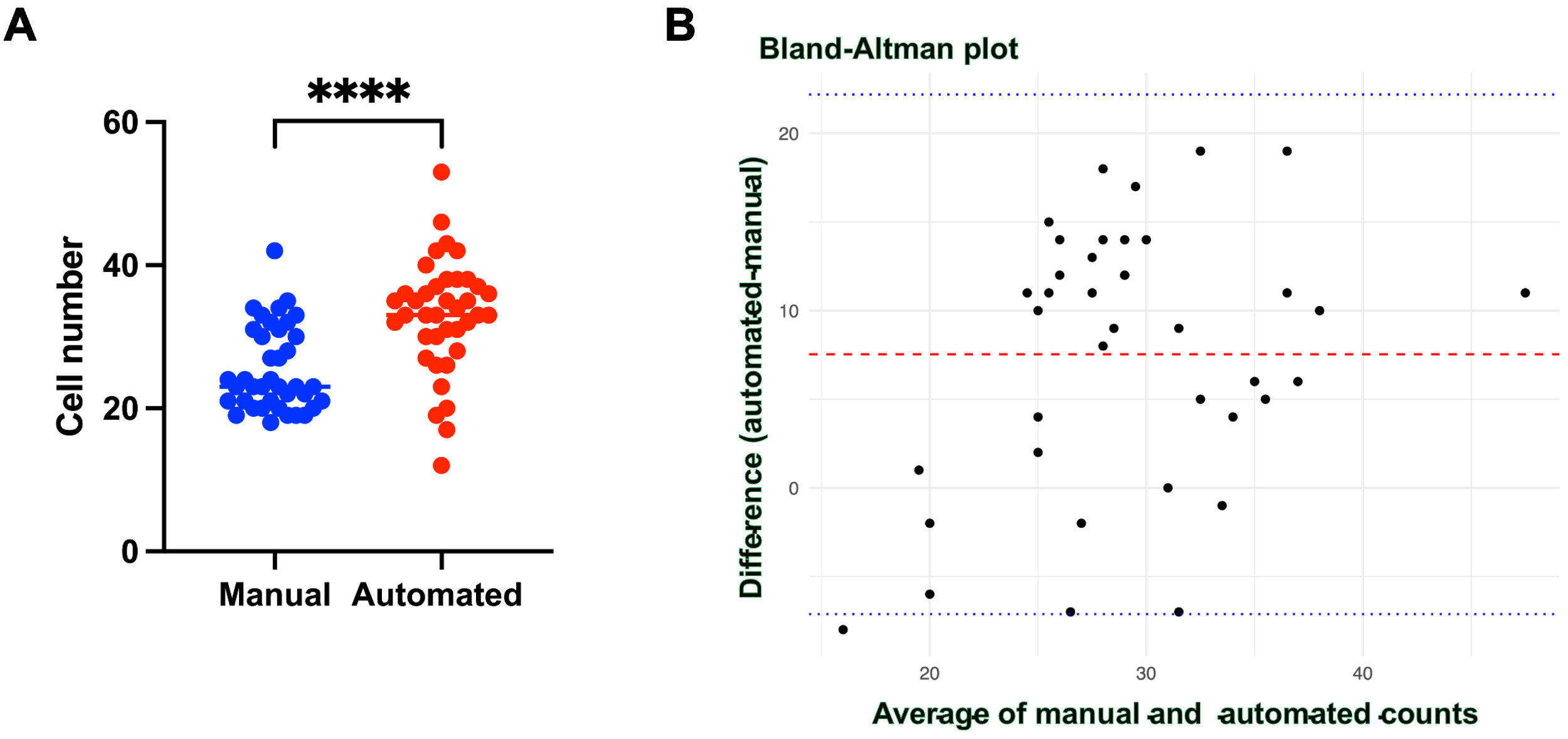


**Fig. S3:** Comparison of manual against automated cell counting. A) Comparison of cell number counted by manual (IMARIS/Fiji) vs automated method (developed connectomics pipeline) for the aged male group. Each point represents the cell count from one sample. (n= 39 cells/group). Statistical significance was determined by the Wilcoxon matched-pairs signed-rank test (p*<0.05, **p<0.01, ***p<0.001, ****p<0.0001). B) Bland-Altman plot displays the differences between cell counts obtained via manual and automated methods against their average. The red dashed line represents the mean difference, while the blue dotted lines indicate the 95% limits of agreement. Each point represents a paired observation from a single sample, with blue points indicating lower average counts and red points higher averages. The plot illustrates the expected discrepancies due to methodological differences, the exclusion of edge cells in manual counting.

**Table S1: Bone structural parameters in the full, trabecular, and cortical regions of the femurs of PolgA mice and WT littermates at 20 and 40 weeks assessed by *ex vivo* micro-CT**

Parameter legend: average volume density (AVD), bone volume fraction (BV/TV), trabecular thickness (Tb.Th), trabecular number (Tb.N), trabecular separation (Tb.Sp), cortical area fraction (Ct.Ar/Tt.Ar), total cross-sectional area inside the periosteal envelope (Tt.Ar), cortical bone area (Ct.Ar), cortical thickness (Ct.Th). Data represent mean±s.d. (n=8-16 mice/group).

|  | **20 weeks** | | | |
| --- | --- | --- | --- | --- |
|  | **Males** | | **Females** | |
|  | PolgA | WT | PolgA | WT |
| **Parameters** |  | | | |
| AVD [%] | 46.05±3.48 | 47.26±2.19 | 46.83±2.05 | 46.74±0.82 |
| BV/TV [%] | 9.05±3.33 | 7.92±1.55 | 2.58±0.94 | 2.62±2.94 |
| Tb.Sp [mm] | 0.34±0.09 | 0.29±0.04 | 0.39±0.10 | 0.39±0.10 |
| Tb.N [1/mm] | 3.16±0.36 | 2.83±0.50 | 2.40±0.47 | 2.39±0.45 |
| Tb.Th [mm] | 0.04±0.00 | 0.06±0.00 | 0.05±0.00 | 0.05±0.00 |
| Ct.Ar/Tt.Ar [%] | 42.38±2.41 | 42.74±1.39 | 47.56±2.01 | 47.25±1.55 |
| Tt.Ar [%] | 2.01±0.20 | 1.95±0.14 | 1.58±0.07 | 1.61±0.12 |
| Ct.Ar [mm²] | 0.85±0.12 | 0.83±0.06 | 0.75±0.03 | 0.77±0.02 |
| Ct.Th [mm] | 0.18±0.00 | 0.18±0.00 | 0.19±0.00 | 0.19±0.00 |

|  | **40 weeks** | | | |
| --- | --- | --- | --- | --- |
|  | **Males** | | **Females** | |
|  | PolgA | WT | PolgA | WT |
| **Parameters** |  |  |  |  |
| AVD [%] | 37.27±3.35 | 46.65±2.53 | 39.29±2.77 | 45.54±1.64 |
| BV/TV [%] | 2.94±1.98 | 6.25±1.62 | 0.53±0.26 | 1.01±0.50 |
| Tb.Sp [mm] | 0.42±0.10 | 0.54±0.13 | 0.93±0.10 | 0.68±0.16 |
| Tb.N [1/mm] | 1.91±0.37 | 2.29±0.54 | 0.99±0.11 | 1.37±0.32 |
| Tb.Th [mm] | 0.04±0.00 | 0.05±0.00 | 0.05±0.00 | 0.05±0.00 |
| Ct.Ar/Tt.Ar [%] | 36.46±2.33 | 40.29±2.24 | 40.95±4.02 | 44.66±2.05 |
| Tt.Ar [%] | 1.91±0.22 | 2.05±0.22 | 1.67±0.10 | 1.69±0.09 |
| Ct.Ar [mm²] | 0.68±0.08 | 0.78±0.04 | 0.69±0.07 | 0.82±0.08 |
| Ct.Th [mm] | 0.15±0.01 | 0.18±0.00 | 0.17±0.02 | 0.19±0.01 |
