# Supplementary material for: Age- and sex-specific deterioration on bone and osteocyte lacuno-canalicular network in a mouse model of premature aging
Source: Bone Res. 2025 May 23;13:55. doi: 10.1038/s41413-025-00428-x (PMC12102221; doi:10.1038/s41413-025-00428-x)
Supplement: Supplementary file 2 — Supplemental Material Videos [file 41413_2025_428_MOESM2_ESM.pptx]

## Slide 1
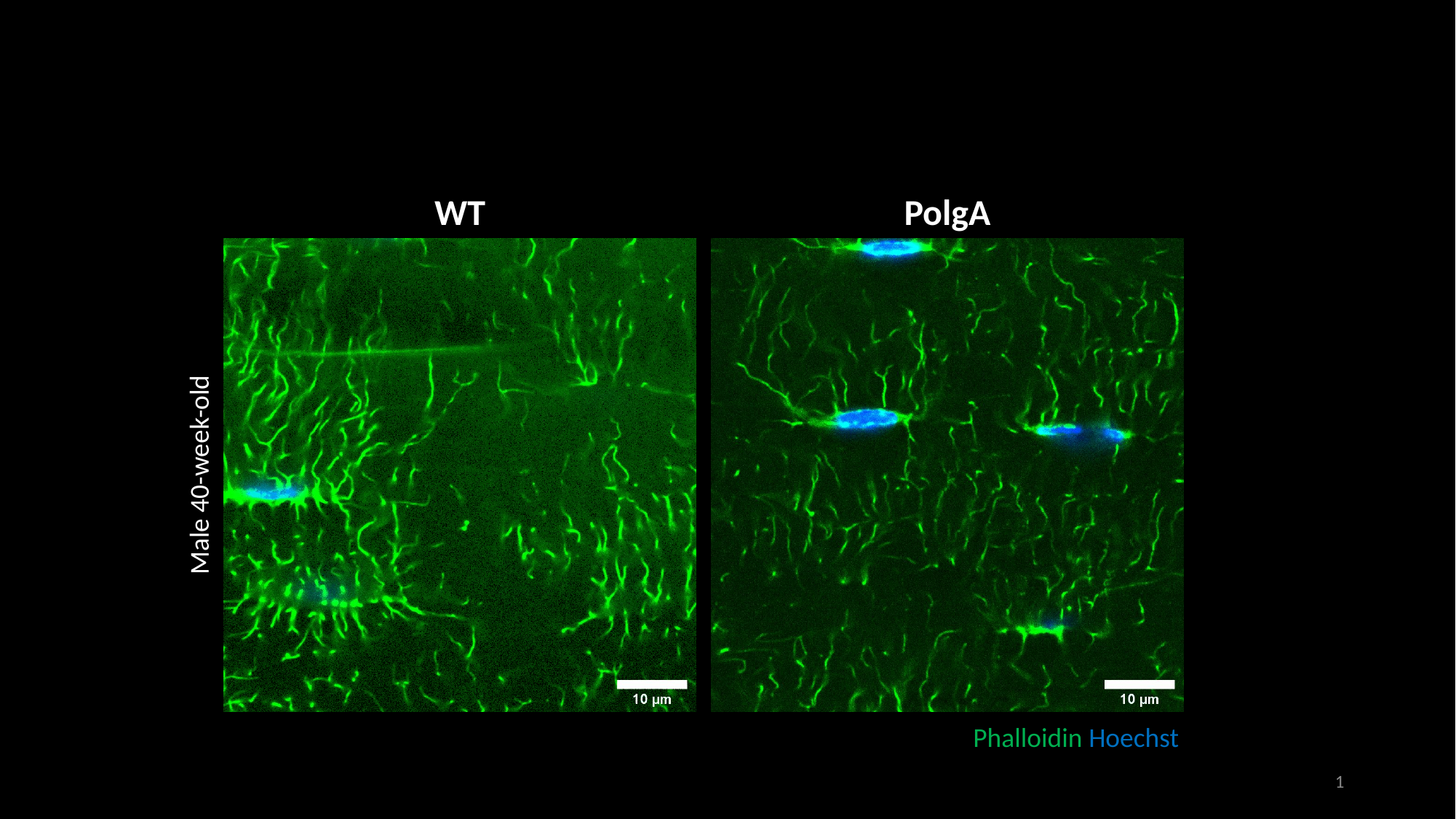

WT
PolgA
Male 40-week-old
Phalloidin Hoechst
1

## Slide 2
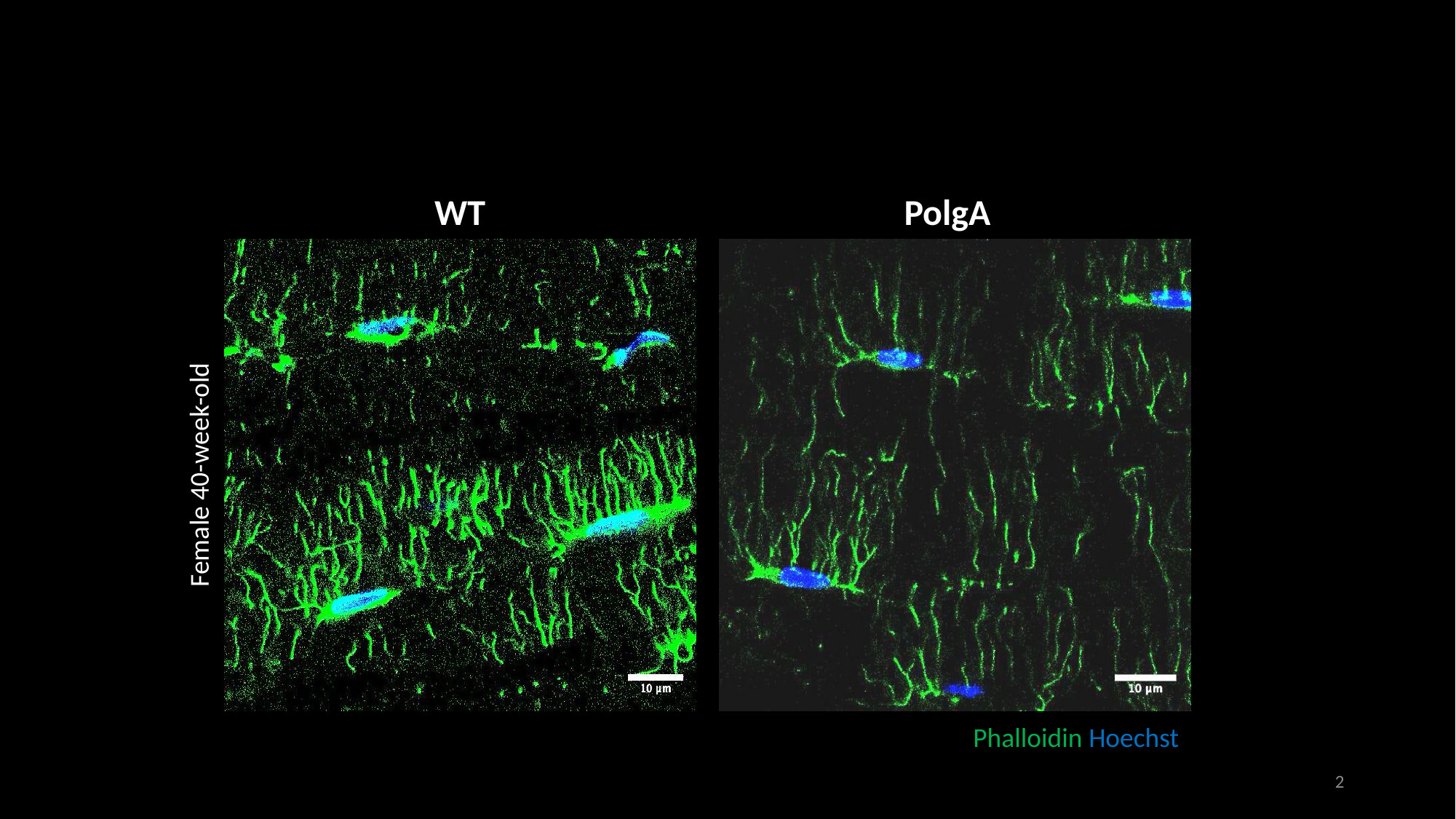

WT
PolgA
Female 40-week-old
Phalloidin Hoechst
2

## Slide 3
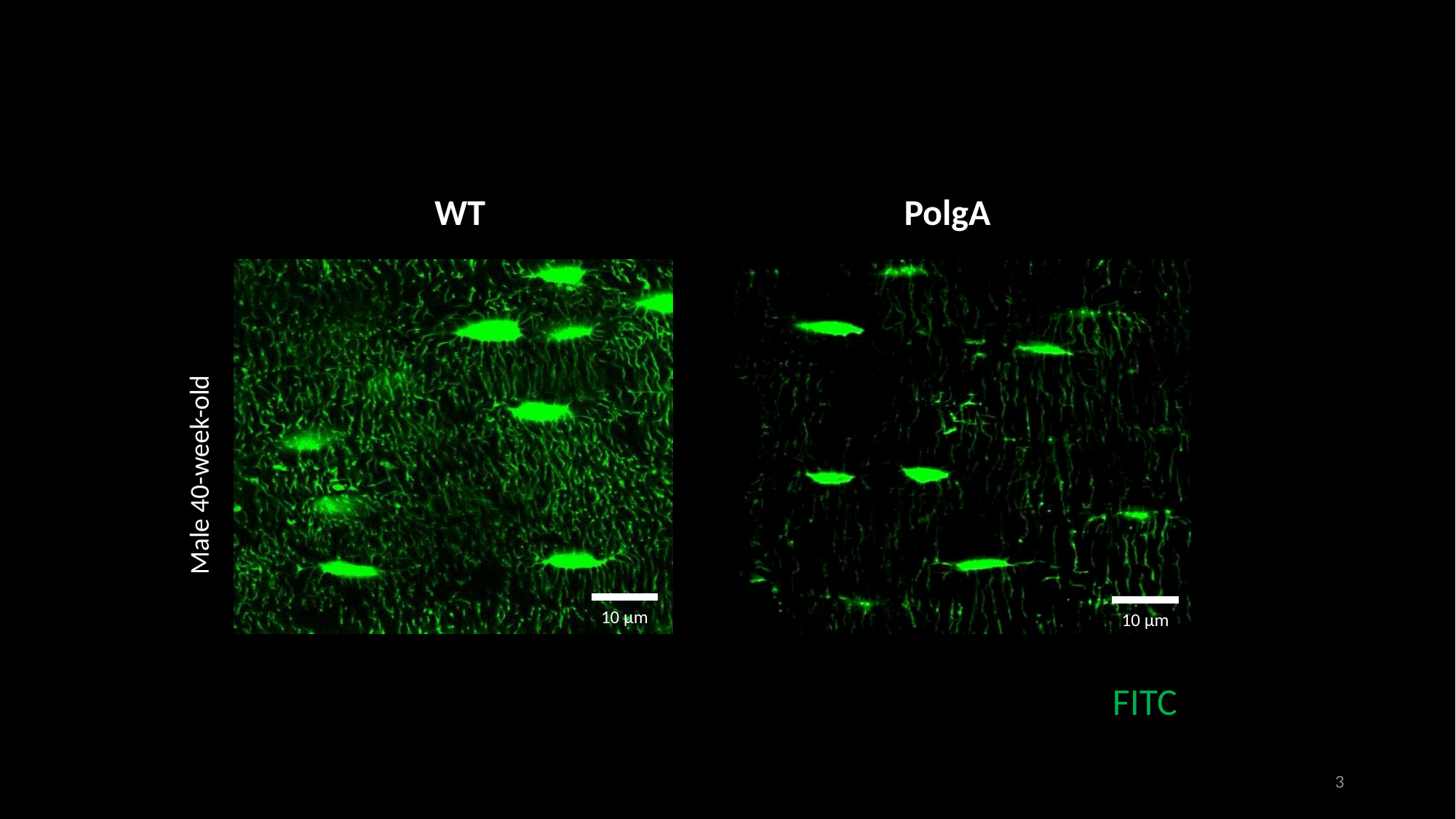

WT
PolgA
Male 40-week-old
10 µm
10 µm
FITC
3

## Slide 4
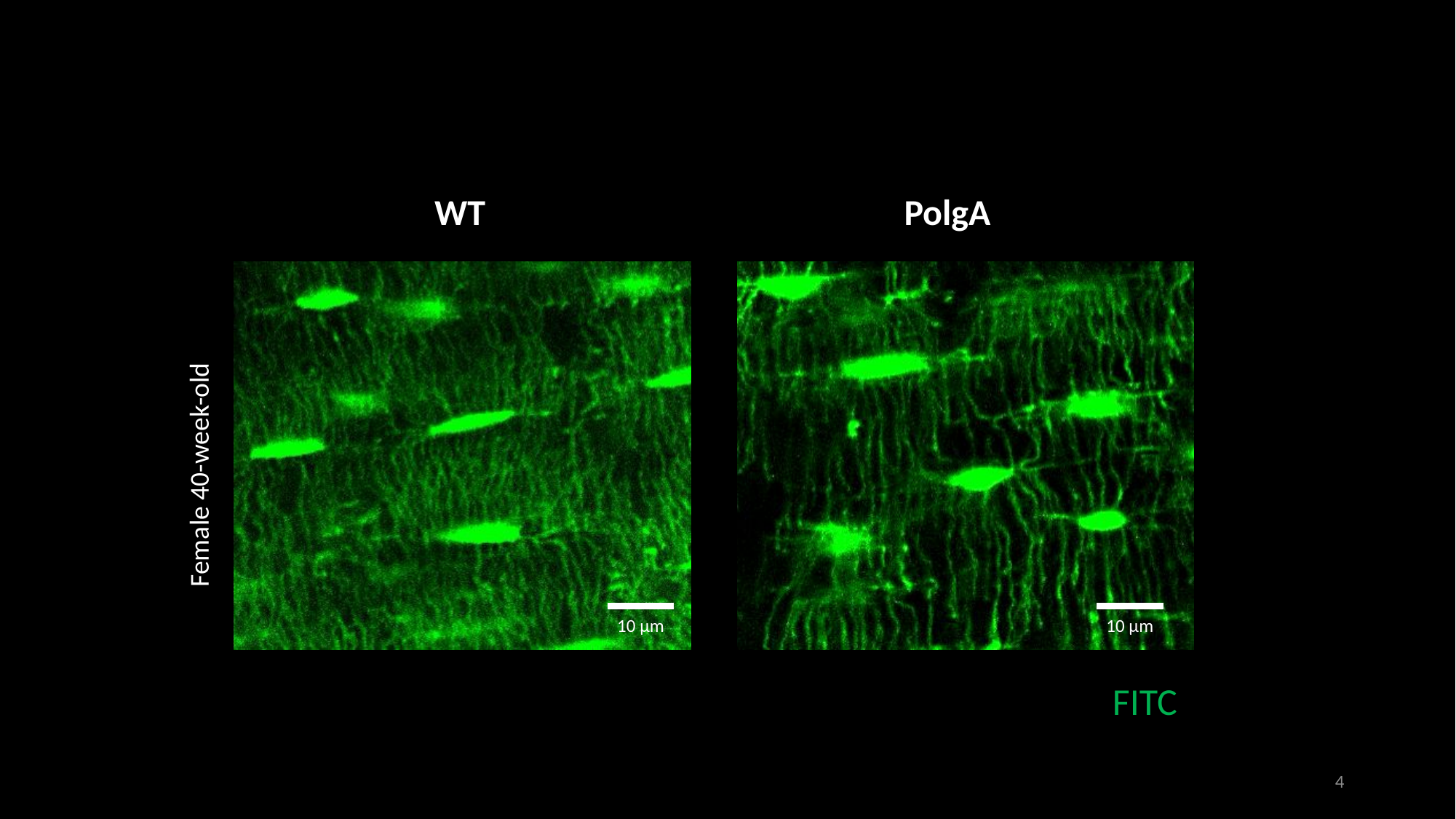

WT
PolgA
Female 40-week-old
10 µm
10 µm
FITC
4
