# Supplementary figures and images for: Age- and sex-specific deterioration on bone and osteocyte lacuno-canalicular network in a mouse model of premature aging
Source: Bone Res. 2025 May 23;13:55. doi: 10.1038/s41413-025-00428-x (PMC12102221; doi:10.1038/s41413-025-00428-x)

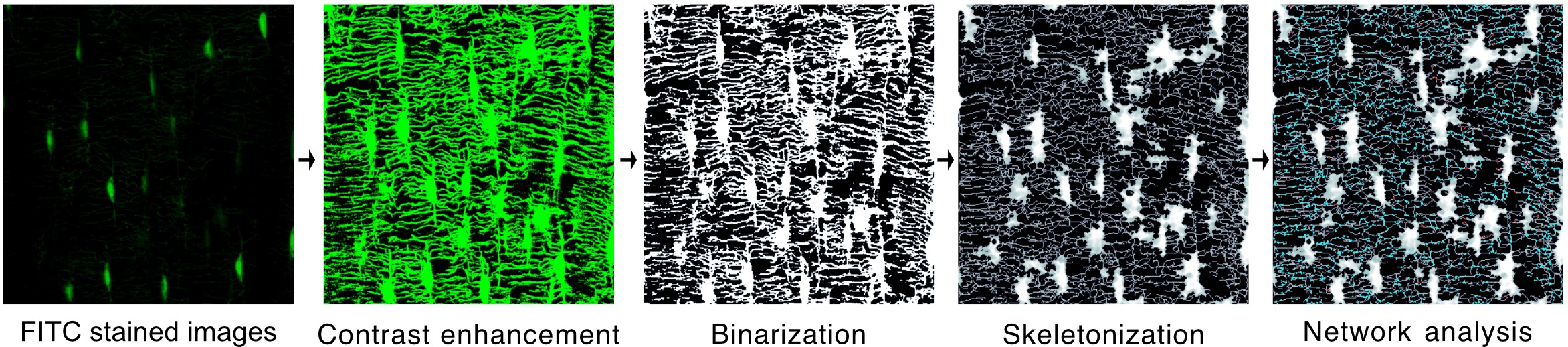

Supplement: Supplementary file 7 — Supplemental Material Python Code [file 41413_2025_428_MOESM7_ESM.zip › ocy_connectomics_preprocessing/assets/LCN_Pipeline.jpg]

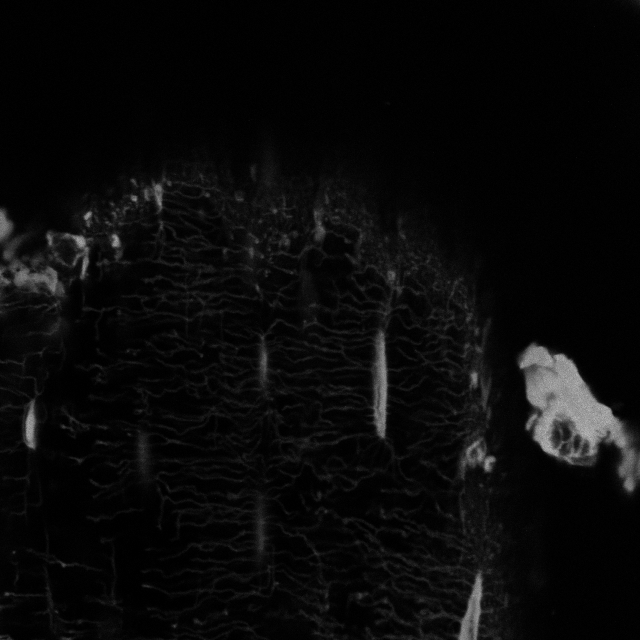

Supplement: Supplementary file 7 — Supplemental Material Python Code [file 41413_2025_428_MOESM7_ESM.zip › ocy_connectomics_preprocessing/data/ExampleSample_ch00.tif]
